# Sensitive Detection of Colorectal Cancer in Peripheral Blood by Septin 9 DNA Methylation Assay

**DOI:** 10.1371/journal.pone.0003759

**Published:** 2008-11-19

**Authors:** Robert Grützmann, Bela Molnar, Christian Pilarsky, Jens K. Habermann, Peter M. Schlag, Hans D. Saeger, Stephan Miehlke, Thomas Stolz, Fabian Model, Uwe J. Roblick, Hans-Peter Bruch, Rainer Koch, Volker Liebenberg, Theo deVos, Xiaoling Song, Robert H. Day, Andrew Z. Sledziewski, Catherine Lofton-Day

**Affiliations:** 1 Department of Visceral-, Thoracic- and Vascular Surgery, University Hospital Carl Gustav Carus Dresden, Dresden, Germany; 2 Semmelweis University, Budapest, Hungary; 3 Department of Surgery, University Hospital Schleswig-Holstein, Campus Lübeck, Lübeck, Germany; 4 Department of Surgery and Surgical Oncology, Charité Campus Berlin Buch, Robert-Rössle-Klinik, Berlin, Germany; 5 Department of Gastroenterology, University Hospital Carl Gustav Carus Dresden, Dresden, Germany; 6 Völklingen Clinic, Völklingen, Germany; 7 Epigenomics AG, Berlin, Germany; 8 Institute of Medical Informatics and Biometrics, Technical University of Dresden, Dresden, Germany; 9 Epigenomics Inc, Seattle, Washington, United States of America; City of Hope Medical Center, United States of America

## Abstract

**Background:**

Colorectal cancer (CRC) is the second leading cause of cancer deaths despite the fact that detection of this cancer in early stages results in over 90% survival rate. Currently less than 45% of at-risk individuals in the US are screened regularly, exposing a need for better screening tests. We performed two case-control studies to validate a blood-based test that identifies methylated DNA in plasma from all stages of CRC.

**Methodology/Principal Findings:**

Using a PCR assay for analysis of *Septin 9* (SEPT9) hypermethylation in DNA extracted from plasma, clinical performance was optimized on 354 samples (252 CRC, 102 controls) and validated in a blinded, independent study of 309 samples (126 CRC, 183 controls). 168 polyps and 411 additional disease controls were also evaluated. Based on the training study SEPT9-based classification detected 120/252 CRCs (48%) and 7/102 controls (7%). In the test study 73/126 CRCs (58%) and 18/183 control samples (10%) were positive for SEPT9 validating the training set results. Inclusion of an additional measurement replicate increased the sensitivity of the assay in the testing set to 72% (90/125 CRCs detected) while maintaining 90% specificity (19/183 for controls). Positive rates for plasmas from the other cancers (11/96) and non-cancerous conditions (41/315) were low. The rate of polyp detection (>1 cm) was ∼20%.

**Conclusions/Significance:**

Analysis of SEPT9 DNA methylation in plasma represents a straightforward, minimally invasive method to detect all stages of CRC with potential to satisfy unmet needs for increased compliance in the screening population. Further clinical testing is warranted.

## Introduction

Colorectal cancer (CRC) is one of the most common neoplasms found in men and women in the United States. The American Cancer Society had estimated that 154,000 new cases of CRC would occur in 2007, resulting in more than 52,000 deaths. Screening programs for the identification of early stage CRC and pre-neoplastic conditions can significantly improve disease outcome because of the treatment benefit of early detection [1]. Current non-invasive screening procedures are not very effective, as they require patient compliance to self-collect stool sample analyzed annually for the presence of occult blood (FOBT) [2]. To date, improvements in feces-based tests by making them more sensitive and more user friendly have not increased compliance in CRC screening. Invasive screening tests such as colonoscopy or sigmoidoscopy, although more effective, require extensive bowel preparation, invasion of privacy, and sedation, and do not overcome current compliance issues in CRC screening. There is growing expectation that the new generation of screening tests based on molecular biomarkers present in blood should improve patient compliance in CRC screening as evidenced by the success of other screening programs such as cholesterol/lipids and prostate specific antigen (PSA) [5]–[7].

Determination of epigenetic events is a strong candidate for early detection of disease since regulation of gene expression by aberrant DNA methylation is a well-characterized event in tumor biology [8], [9], and is extensively described for CRC [10]–[12]. Increased levels of free-circulating methylated DNA in the blood of cancer patients compared to healthy controls have been reported [13], [14]. Several laboratories also reported promising DNA methylation-based marker candidates for detection of CRC [15]–[19]. Translating such marker candidates into clinically validated and commercially viable tests has been exceedingly slow and inadequate. To facilitate improvements in biomarker translation medicine Pepe *et al.* proposed a systematic process for biomarker validation for early detection of cancer with 5 distinct phases, each phase providing increased level of evidence of marker validation [20]. In an initial study we presented the first level of evidence that SEPT9, a DNA methylation-based biomarker, effectively discriminates CRC from normal specimens [21]. The Septin 9 gene belongs to a class of GTPases involved in numerous cellular process [22].The gene has been shown to have multiple alternatively spliced transcripts encoding at least 5 characterized polypeptides designated v1–v5 [23], some of which have been associated with cancer. The ratio of v4 and v4* expression, identical proteins encoded by different transcripts, has been shown to be altered in ovarian cancer with v4 being the predominant form expressed in normal cells and v4* expressed in tumors [24]. Recent studies of the v1 isoform suggest that over-expression of this polypeptide may promote tumor progression in mammary tissue [25]. Our previous work describing aberrant methylation in the promoter region of the v2 transcript indicates that methylation in this region is associated with colorectal cancer [21]. Moving forward in the process proposed by Pepe *et al*, in this report we provide the second level of evidence by presenting results from validation of clinical assay for SEPT9 in two large independent plasma sets demonstrating the potential of this marker for early detection of CRC. Increasing the number of assay replicates tested resulted in high sensitivity for CRC with excellent specificity in healthy controls. Specificity was further evaluated in a number of disease controls. Finally, the SEPT9 methylation of pre-malignant lesions is reported.

## Methods

### Patients

700 patient samples collected at 9 sites were measured in the training study. It included patients with all stages of colorectal cancer, individuals without diseases of the colon as verified by colonoscopy, additional disease controls and a number of patients with adenomatous polyps (Table 1). A subset of 354 samples (only CRCs and normals with complete SEPT9 measurement) was used to generate the training set algorithm. The test study consisted of 547 patient samples (a subset of 309 CRCs and normals with complete SEPT9 measurement was used as a test set) collected at 14 sites and included similar clinical categories of samples as used in the training study. Additional disease controls were collected from individuals with non-colorectal cancers and various non-cancerous diseases for additional specificity testing. Not all of those subjects were verified with colonoscopy as CRC-and/or adenoma-free. Written informed consent was obtained from all study participants adhering to the local ethical guidelines.

**Table 1:** Patient characteristics of training and test studies.

Diagnosis Group	Description	Total 4	Gender		Age					Location 1	
			Male	Female	<50	50–59	60–69	≥70	Median^5^	Distal	Proximal
**Training Set**											
Colorectal Cancer	Stage I	63	30	33	9	16	18	20	61	41	21
	Stage II	83	46	37	10	28	28	17	61	50	33
	Stage III	59	37	22	2	22	26	9	60	47	10
	Stage IV	29	16	13	5	8	10	6	60	21	6
	Stage NA	19	15	4	2	5	8	4	64	14	5
Adenomas/Polyps	<10 mm	53	27	26	10	13	16	14	61	34	19
	≥10 mm	65	37	28	38	25	2	0	47	62	3
Healthy/Normal Colon		102	36	66	19	34	27	22	59		
Non-colorectal Cancer^2^		60	34	25	11	18	20	11	61		
Non-cancerous Disease 3		167	63	103	71	39	38	19	52		
Total		700	341	357	177	208	193	122	58	269	97
**Test Set**											
Colorectal Cancer	Stage I	22	15	7	0	4	6	10	69	11	1
	Stage II	37	23	14	1	8	13	11	67	11	8
	Stage III	54	27	27	2	10	17	20	67	26	7
	Stage IV	11	8	3	0	4	4	3	63	4	0
	Stage NA	3	3	0	1	1	0	0	49	1	0
Adenomas/Polyps	<10 mm	34	21	13	0	4	19	11	68	0	0
	≥10 mm	17	14	3	1	4	9	3	66	0	0
Healthy/Normal Colon		184	76	106	34	72	42	33	56		
Non-colorectal Cancer		36	22	14	4	5	14	8	65		
Non-cancerous Disease		149	57	92	14	29	56	41	66		
Total		547	266	279	57	141	180	140	63	53	16

1 Colorectal lesions are divided regarding their location into ‘Distal’: descending, sigmoid, left flexure, rectum and ‘Proximal’: cecum, ascending, right flexure, transverse.

2 Non-colorectal Cancer include bladder, breast, liver, lung, prostate and pancreatic cancers.

3 Non-cancerous Diseases include gastritis, hypertension, type II diabetes, liver disease, rheumatoid and non- rheumatoid arthritis, cholycystitis, cystitis, chronic heart disease, esophagitis, IBD, pancreatitis, chronic renal failure, respiratory infection.

4 Total sample numbers can be higher than the sum of all subgroups when sample annotations are missing, for some patient samples SEPT9 methylation measurement was incomplete.

5 Median patient age at time of sample collection in years.

All cancer patients and healthy controls enrolled in both studies were at least 40 years old with a preference for patients 50 and older and derived predominantly from the same clinics. All subjects participating had neither a personal history of HIV, HBV or HCV or previous history of cancer with the exception of basal cell carcinoma nor symptoms of severe acute nor exacerbated chronic disease.

Blood from all subjects has been drawn either before or more than 2 days and up to 6 months after colonoscopy and prior to starting any cancer specific treatment. Cancer diagnosis was confirmed histologically from the surgical specimen and only adenocarcinomas were included in this study.

### Sample processing workflow

**Figure 1:**
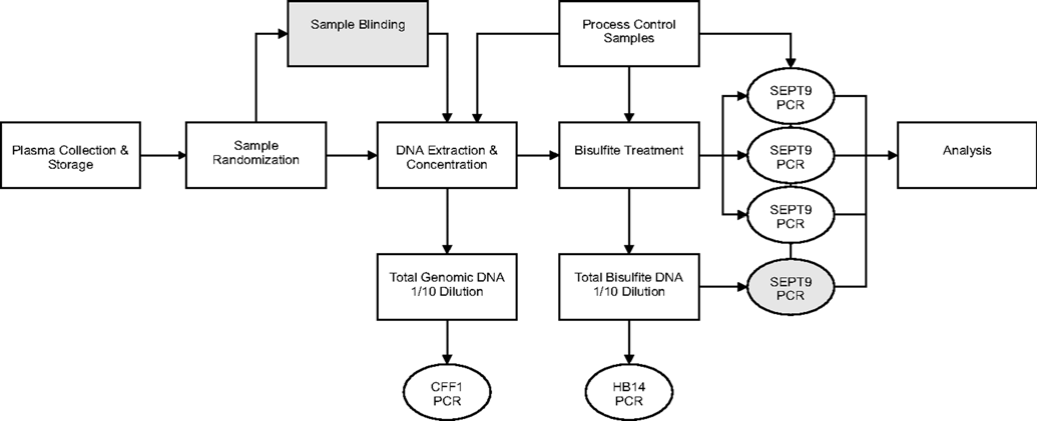
Sample processing workflow. The diagram depicts the major sample processing and laboratory workflow steps. It shows where process control samples are introduced into the workflow and what assays were used to measure output of each process step. Grey boxes indicate test set specific workflow steps.

A 3-part workflow was developed for the SEPT9 test (Figure 1). DNA Extraction: Free-floating circulating DNA was extracted from plasma using the Total Nucleic Acid Large Volume DNA extraction kit (Roche Applied Science, Indianapolis, IN) and Roche MagNaPure device. Eight or 16 mL of plasma was distributed in MagNaPure wells (1 mL per well), extracted following the kit protocol, and DNA was eluted in 100 µL aliquots. Eluates from each patient were pooled and concentrated to a final volume of 100 µL using Microcon YM-30 filters (Millipore). A 5 µL aliquot of each sample was retained to measure total genomic DNA using the real-time PCR assay (CFF1) described in Supplementary Table S1. Bisulfite Treatment: Concentrated DNA samples were bisulfite treated using methods to achieve maximal conversion and DNA recovery [35]. A 5 µL aliquot of each sample was retained to measure total bisulfite treated DNA using the real-time PCR assay (HB14) described in Supplementary Table S1. Real Time PCR Analysis: The SEPT9 real time PCR assay is designed surrounding the transcription start site of the v2 transcript. The real time assay sequences, cycling conditions and quality control process are described in the Supplementary Text S1 and Supplementary Figure S1. For total genomic and total bisulfite converted DNA measurements, PCR was performed on a 1∶10 dilution (in elution buffer) of the respective materials. For SEPT9 measurements on samples, reactions were performed on 10–12.5 µL of undiluted DNA depending on experiment. PCR was performed using the Roche LightCycler 2.0 device with the FastStart DNA Master HybProbe master mix (Roche Applied Science). Each PCR run included a DNA Standard curve prepared using bisulfite treated CpGenome Universal Methylated DNA (Millipore (Chemicon), Billerica, MA) at concentrations between 50 pg/rxn–20 ng/rxn. Each run also included a no template control which was left uncapped throughout the process to control for contamination, and 3 no-template controls which were used to establish the baseline of the PCR reaction. Samples were run as single capillaries on each PCR run, and replicates were performed in separate PCR runs. Amplification curves for each reaction were manually verified by 2 independent reviewers.

### Data analysis

Patient samples for training and test study were grouped according to diagnosis and gender and randomly assigned to DNA extraction batches. In addition test study samples were blinded prior to processing in the laboratory and data analysis. Quantitative real-time PCR analysis of plasma samples was performed as described earlier and replicate measurements were averaged.[21] Chi square test was used to compare detection rates between different locations of tumor, age, tumor stage and gender. Wilcoxon-Mann-Whitney test and Kruskall-Wallis test were used to quantitatively compare methylated Septin 9 DNA concentrations between different locations of tumor, age, tumor stage and gender. Confidence intervals for proportions of detected samples were set at 95% and based on binomial distributions. All P values are two-sided.

## Results

The objective of this investigation was to validate the use of SEPT9 hypermethylation as a biomarker for colorectal cancer by determining the optimal classifier using a set of samples (training study) and confirming the selected classifier in an independent sample set (test study). Each plasma sample was processed using a three-step workflow outlined in Figure 1. The workflow process was carefully monitored with addition of positive and negative controls at each process step and individual samples were accepted for final analysis after passing quality control specifications (see Supplementary Text S1).

### Training Study

**Figure 2:**
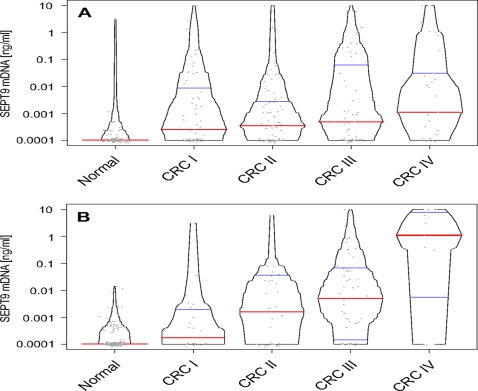
Concentrations of methylated SEPT9 DNA in plasma. Box-percentile plots of training set methylated SEPT9 DNA concentrations in plasma are shown for colonoscopy-verified normal patients (Normal) and patients with colorectal cancer (CRC). Median DNA concentrations are red horizontal lines; 25th and 75th percentiles are blue horizontal lines. The width of the box-percentile plot at any given height is proportional to the percent of observations that are more extreme in the direction leading away from the median. Individual measurement values are plotted as grey circles. B) Box-percentile plots of testing set methylated SEPT9 DNA concentrations in plasma.

The demographic and clinical parameters of the subjects included in the training study are outlined in Table 1. Three hundred fifty-four colorectal cancer and normal control samples were included in the primary data analysis. The training study data was analyzed qualitatively, where a SEPT9 reaction was called positive if a distinct PCR curve was detected. Each plasma sample was measured in three independent PCR reactions and based on analysis of clinical performance, patient samples were classified as positive if two out of three PCR replicates were called positive. Based on this qualitative algorithm we measured a sensitivity of 48% for colorectal cancer and a specificity of 93% for non-colorectal disease colonoscopy-verified samples. Alternatively, we determined optimal thresholds based on quantitative analysis of the SEPT9 marker. These quantitative algorithms did not improve overall marker performance (see Supplementary Table S2). Using the qualitative analysis, we analyzed correlations between detection of colorectal cancer and different clinical parameters. There was no significant difference in detection rate by location of tumor, age or gender. Early stage colorectal cancers were detected at slightly lower rates (43%) than later stage cancers (55%) but the difference was not significant. There was also no significant quantitative difference between amounts of methylated SEPT9 DNA between different stages (Figure 2A). The SEPT9 marker also detected 22% of polyps larger than 1 cm.

### Test Study

To confirm the clinical performance of the SEPT9 training algorithm we collected plasma samples from an independent patient set (test set – Table 1). Compared to the training set the test set contained significantly fewer early stage cancers and cancer patients were slightly older. Using the SEPT9 threshold determined in the training study we were able to confirm marker performance in the test set of 309 colorectal cancer patients and healthy controls with sensitivity of 58% and specificity of 90% (Table 2).

**Table 2:** Septin9 marker performance – training and testing studies.

Patient Group	Septin9 Training			Septin9 Testing			Septin9 Testing Re-Analyzed		
	2 positive out of 3			2 positive out of 3			2 positive out of 3 OR positive diluted		
	%	[95% CI]	Pos/Total	%	[95% CI]	Pos/Total	%	[95% CI]	Pos/Total
Healthy	7	[3,14]	7/102	10	[6,15]	18/183	10	[6,16]	19/183
CRC All	48	[41,54]	120/252	58	[49,67]	73/126	72	[63,80]	90/125
CRC I–III	47	[40,54]	95/204	56	[47,66]	63/112	70	[61,79]	78/111
CRC I	43	[30,56]	27/63	36	[17,59]	8/22	50	[28,72]	11/22
CRC II	44	[33,55]	36/82	56	[38,72]	20/36	69	[52,84]	25/36
CRC III	54	[41,67]	32/59	65	[51,77]	35/54	79	[66,89]	42/53
CRC IV	55	[36,74]	16/29	73	[39,94]	8/11	91	[59,100]	10/11
Polyp≥10 mm	22	[13,34]	14/64	18	[4,43]	3/17	18	[4,43]	3/17
Polyp<10 mm	9	[3,21]	5/53	9	[2,24]	3/34	9	[2,24]	3/34

There was no significant difference in detection rate by location of tumor, age or gender. Early stage colorectal cancers were detected at lower rates (36%) than later stage cancers (73%) but the difference in detection rate was not significant. However, there was a significant quantitative difference between amounts of methylated SEPT9 DNA between different stages (P<0.002; Figure 2B). Large polyps (>1 cm) were detected at a rate of 18%.

### Improved process yields better SEPT9 performance

**Figure 3:**
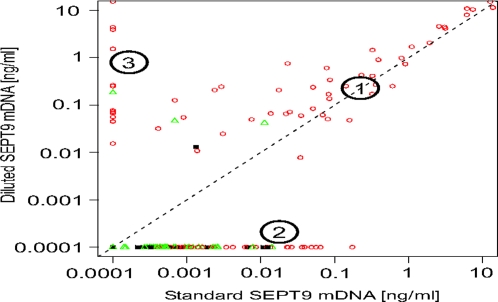
Correlation between standard and diluted SEPT9 measurement in testing set. X-axis – concentration of SEPT9 in standard test set samples (mean of three standard replicates) Y-axis – concentration of SEPT9 in 1∶10 diluted test set samples (single replicate). mDNA – methylated DNA. Group 1 – samples with the same SEPT9 amplification in standard and diluted concentration, group 2 – samples with SEPT9 amplification only in standard concentration, group 3 – samples with SEPT9 amplification only in diluted concentration. Green triangles – healthy colon, red circles – colorectal cancer, black squares – polyps.

During our routine quality control we observed sporadic inhibition of the PCR reaction (see Supplementary Figure S2). Therefore we analyzed an additional SEPT9 replicate using 10 fold-diluted samples available from the original total bis-DNA measurements. The correlation plot of the standard SEPT9 measurement (mean of three standard replicates) and a single measurement of the diluted sample is shown in Figure 3.

There are three groups of samples: group1 in which SEPT9 amplification was identical in standard and diluted samples, group 2 in which only standard concentration samples amplified and group 3 in which only the diluted samples amplified. Group 2 consists of samples for which no PCR inhibition was observed, but contained levels of tumor DNA insufficient for amplification of SEPT9 after dilution. Group 3 samples displayed PCR inhibition in standard concentration, but when diluted, showed SEPT9 amplification. We incorporated the result of this additional measurement into a new algorithm in which a patient sample is classified positive if either two out of three standard PCR replicates are positive (training set algorithm) OR the measurement of the diluted sample is positive. Reanalysis of the test set results using this algorithm dramatically improved the performance of the SEPT9 assay (Table 2).

Overall colorectal cancer detection reached 72% while maintaining very high specificity of 90%. Especially impressive were improvements in detection of early stage cancer (Stage I/II – sensitivity 62%) confirming the potential value of SEPT9 as an early detection biomarker for colorectal cancer.

### Non colorectal cancer (NCC) and non cancerous diseases (NCD) sample analysis

We also collected and analyzed a subset of plasma samples from patients diagnosed with non-colorectal cancers (NCC) including bladder, breast, lung, liver, pancreas and prostate cancers as well as non-cancerous disease that may have confounding effects on the SEPT9 marker performance. As shown in Table 3, SEPT9 is exceptionally specific regarding colorectal cancer when compared to detecting NCCs: only a few plasma samples from liver cancer (2 out of 8 samples), lung cancer (4 out of 13) and bladder cancer (4 out of 19) patients were positive for the marker. The low percentage of SEPT9 present in multiple non-cancerous disease groups, e.g. chronic renal failure, gastritis, and chronic respiratory infections (see Table 3) points to its high specificity for colorectal cancer malignancy.

**Table 3:** Septin9 performance in NCC and NCD samples.

Patient Group	Positive/Total
***Non Colorectal Cancer***	**11/96**
Bladder	4/19
Breast	0/30
Liver	2/8
Lung	4/13
Pancreas	0/5
Prostate	1/19
Stomach	0/2
***Non Cancerous Disease***	**41/315**
Inflammatory Bowel Disease	2/20
Esophagitis	1/28
Gastritis	7/24
Diverticulitis	0/5
Diverticulosis	0/9
Appendicitis	1/8
Pancreatitis	1/20
Cholycystitis	3/21
Cystitis	1/13
Liver Disease	1/14
Collagenosis	0/5
Diabetes Type II	0/14
Lupus	2/14
Allergy	0/5
Arthritis non-rheumatoid	2/28
Arthritis rheumatoid	4/25
Heart Disease Chronic	2/17
Pyelonephritis	3/8
Renal Failure Chronic	5/13
Respiratory Infection	6/24

## Discussion

In this report we determined performance of a real-time PCR assay for methylated SEPT9 DNA first in a training study and then in a blinded independent testing study. Overall sensitivity for colorectal cancers was comparable in both studies as identified using the original training algorithm. Sensitivity increased to 72% with an additional SEPT9 replicate of the 10-fold diluted plasma sample. The marker was shown to be sensitive for early-stage colorectal cancers identifying 62% of Stage I/II CRCs. There was a trend for early stage colorectal cancers to be detected at slightly lower rates than later stage cancers in both the training and testing study, but the differences were not significant due to insufficient number of samples for each individual stage of cancer. Sensitivity of CRC detection was completely independent of tumor location in colon. This may be a general feature of methylation biomarkers since Chen *et al.* noted a similar lack of correlation to stage or location in a recent study in stool of patients with colorectal cancer [26]. Other fecal tests such as FOBT and iFOBT have been shown to have a decreased sensitivity for both proximal colorectal cancers and early stage cancers [27]. Finally, our results indicate that the biomarker is also highly specific (90–93%) in healthy individuals.

We developed a very sensitive PCR assay to detect methylated v2 promoter of SEPT9 in plasma samples. Effective validation of such a test depends as much on quality of the biomarker as on the quality and consistency of the workflow steps, including the final marker measurement. We controlled our workflow at each step; assessing variability of DNA extraction, bisulfite conversion and PCR amplification and because of such quality control we were able to identify sporadic PCR inhibition in the test set. Overcoming this issue by diluting the PCR reaction 10-fold and combining this measurement with the undiluted measurement yielded the true performance of the SEPT9 marker. The use of four assay replicates (3 standard replicates and 1 diluted) allows alternative analysis focusing either on sensitivity or specificity of the marker assay. By requiring SEPT9 to be present in all standard replicates or one diluted replicate before a sample is classified positive, the test achieves a very high specificity (97%) and still retains substantial sensitivity (65%). When only 1 of 4 replicates (either one standard or one diluted) is required to be positive for a positive call (high sensitivity mode) the sensitivity increases to 77% whereas specificity is still respectable at 75% [see Supplementary Table S3].

The control patient groups in our studies included a primary set of individuals without diseases of the colon as verified by colonoscopy but some with conditions likely to be present within the CRC screening population. We made every effort to avoid patient selection bias in the study by requiring controls to be in the same age range as CRCs and to be derived predominantly from the same clinics as our CRC patients. Despite these efforts some biases remained: the majority of CRC patients in training and test set are male (57% and 60% respectively), and test set patients are slightly older than training set patients. However, since we found that detection of CRC is independent of age and gender the reported SEPT9 performance should be unaffected. Our primary analysis compares the performance of the marker in plasma from patients with colorectal cancer vs. the subjects without colorectal disease. We performed additional analyses using non-cancerous disease controls and plasma from cancers other than colorectal to identify potential specificity issues. The numbers of patients with these conditions are not weighted according to their prevalence, and therefore true specificity regarding these diseases cannot be determined for the target screening population. Since the observed positive rate of the marker is very low in the control samples, we believe that the SEPT9 marker will perform well in future prospective clinical studies when measured in CRC screening population.

The SEPT9 test aims at detecting asymptomatic colorectal cancer cases. We also obtained preliminary data on polyp detection with the SEPT9 assay that indicate some pre-malignant changes are identified with our test. The ultimate CRC screening test should also target adenomas that will advance to cancer and that could be removed during follow up colonoscopy. While today we do not yet fully understand the natural history of adenomas, and cannot predict which ones would progress to cancer, it is tempting to speculate that epigenetic markers, like SEPT9, may help with such polyp stratification.

The *Septin 9* gene, also called MSF, encodes a mammalian septin protein involved in many cellular processes. Disruption of the action of *Septin 9* results in incomplete cell division [28]. *Septin 9* and other proteins have been shown to be fusion partners of the proto-oncogene *MLL* suggesting a role in tumorigenesis [29], [30]. *Septin 9* has also been shown to be in a frequently deleted region in breast and ovarian cancers in loss of heterozygosity (LOH) studies, a finding that further implicates the gene as a possible tumor suppressor [31]. Burrows *et al.* reported an in depth study of expression of the multiple isoforms of the *Septin 9* gene in ovarian cancer and showed tissue specific expression of various transcripts [32]. Expression of the v4 transcript of *Septin 9* was shown to be absent or diminished in several cell lines and could be reactivated by treatment with 5-azacytidine, providing initial evidence of potential regulation of the gene by DNA methylation. Additional evidence of methylation control of the v2 transcript was provided in a recent quantitative analysis in tissues and plasma from colorectal cancer patients [21]. Furthermore a recent study by Bennett *et al.* indicates that methylation of the Septin 9 gene also occurs in head and neck cancers, however the region of the methylation event is not described [33]. Over-expression of *Septin 9* isoforms has also been demonstrated in a number of tumor tissues. A previous study of over 7000 normal and tumor tissues indicates that tissue specific expression of Septin 9 transcripts occurs in a wide variety of cancers [23] The authors speculate that the gene is likely a type II cancer gene where changes in RNA transcript processing control regulation of different protein products, and the levels of these altered protein isoforms may provide answers to the gene's role in malignancy. This hypothesis is consistent with recent studies that show Septin 9 isoform over-expression is associated with an oncogenic phenotype [24], [25] and evidence that Septin 9 v1 over-expression is associated with tumor resistance to drugs that disrupt microtubule formation [34].

So far current CRC screening strategies failed to improve patient compliance and to lower mortality of colorectal cancer. Most experts anticipate an improved patient CRC screening compliance with blood-based test, as was the case for prostate cancer screening with PSA or in heart disease with cholesterol/lipids testing. This report describes the first validation of a plasma-based DNA methylation test performed in two large well-controlled case-control studies confirming clinical potential of SEPT9 biomarker for CRC screening application. We believe that such an easily administered blood-based test for early detection of colorectal cancer followed by colonoscopy for positive individuals has the potential to be a very effective tool for reducing mortality from this disease. The SEPT9 marker assay warrants further evaluation as a test for early CRC detection and prospective studies are planned to determine clinical performance in screening guideline-eligible screening populations.

## Supporting Information

Text S1 Supplementary methods
(0.03 MB DOC)

Table S1 Primer, probe and blocker sequences of the marker SEPT9 real-time PCR assay, and the control CFF1 and HB14 real-time PCR assays
(0.02 MB DOC)

Table S2 SEPT9 marker performance in training set - alternative algorithms.
(0.02 MB DOC)

Table S3 SEPT9 marker performance in test set - alternative analysis
(0.03 MB DOC)

Figure S1 Shewhart control charts of total genomic DNA recovery (upper) and SEPT9 marker DNA (lower) for processing controls in the training set.
(0.08 MB DOC)

Figure S2 Shewhart control charts of total genomic DNA recovery (upper) and SEPT9 marker DNA (lower) for processing controls in the test set.
(0.08 MB DOC)
