# Supplementary material for: Sensitive Detection of Colorectal Cancer in Peripheral Blood by Septin 9 DNA Methylation Assay
Source: PLoS One. 2008 Nov 19;3(11):e3759. doi: 10.1371/journal.pone.0003759 (PMC2582436; doi:10.1371/journal.pone.0003759)
Supplement: Text S1 — Supplementary methods (0.03 MB DOC) [file pone.0003759.s001.doc]

# SUPPLEMENTARY TEXT S1

# Patients and Methods

## *Collection and preparation of plasma samples-*

##

Blood was obtained by intravenous collection using EDTA Vacutainer™ tubes (purple top - Becton/Dickinson). Plasma was separated within 4 hours post blood draw by centrifugation at 1500 X g for 10 minutes followed by careful removal of plasma without disturbing the buffy coat. Plasma was then placed in a 15 ml tube and again centrifuged at 1500 X g for 10 min. If more than one tube was obtained per patient, plasma was pooled before aliquoting in cryovials for storage at -80^o^C.

The realtime assay for Septin 9 was developed based on Heavy Methyl technology in which oligonucleotide primers were designed for bisulfite converted sequences using methylation non-specific primers (containing no CpG sites), methylation specific probes and blocker sequences specific for unmethylated DNA to inhibit amplification of undesired sequence. The assay was performed on the Roche LC 2.0 using the FastStart Kit. Following a 10 minute activation step at 95^o^C, a 3-step cycling process (95^o^C for 10 sec, 56^o^C for 30 sec and 72^o^C for 10 sec) was implemented for 50 cycles.

The CFF1 total genomic DNA assay and HB14 total bisulfite assay were also run on the LC 2.0 with the FastStart kit. Cycling conditions for the CFF1 and HB14 assays were a 10 min activation step at 95^o^C followed by a 45-cycle 2-step amplification process. For CFF1 the amplification conditions were 95^o^C for 10 sec and 58^o^C for 60 sec. For HB14 amplification conditions were 95^o^C for 10 sec and 60^o^C for 45 sec.

**Processing Quality Control:**

Assay Quality Control: Oligonucleotide primers, blockers and probes were synthesized by IDT, Biomers and TIB Molbiol respectively. Prior to the studies, oligonucleotide batches were analyzed by mass spectrometry to ensure quality. The 90% limit of detection (LOD) of each assay was determined by a dilution series of methylated (Sss1 treated) DNA in a background of 50 ng blood DNA (human genomic DNA, Roche Applied Science). For the SEPT9 assay, LOD results were based on 3 different lots of pooled Peripheral Blood Leukocyte DNA as background. At least 18 replicate assays were tested for each dilution and PBL lot. The 90% LOD was defined as the lowest concentration of spiked methylated DNA in a background of 50 ng human genomic DNA for which the measurement values had an area under theROC curve (AUC) of_0.9 compared with measurements without spiked methylated DNA. Assays included in the study had LOD measurements below 25 pg.

Processing Controls: The positive control for DNA extraction consisted of 5% BSA into which was spiked 25ng/mL of CpGenome Universal Methylated DNA (Chemicon). The extraction negative control was a 5% BSA solution. The positive control for bisulfite treatment was a 100 μl solution of a 1:10 mixture of CpGenome Universal Methylated DNA **:** PBL DNA at a final concentration of 1 ng/ul. The negative control for bisulfite treatment was a 10 mM Tris solution. Positive and negative processing controls were run prior to the start of the study to establish control parameters, and included with each extraction and bisulfite treatment batch to ensure the process remained in control.

All samples that contained no measurable bis-DNA as well as samples that failed to generate at least two valid measurements of Septin 9 were excluded from analysis. We observed no amplification of our negative controls confirming earlier observations that PCR contamination is not an issue in our sample processing. We were also able to detect (using our positive control quality system) a faulty batch of MagNaPure beads that lead to significantly lower DNA extraction yields in a series of extraction batches (Figure S1) in the training set. When the faulty batch was replaced, DNA extraction yields returned to normal. Quality of the bisulfite conversion process was established by correlation of genomic DNA input and bisulfite DNA yield. This high degree of correlation supports our observation that the bisulfite treatment method results in an optimal conversion and little loss of DNA.
