# Supplementary material for: Sensitive Detection of Colorectal Cancer in Peripheral Blood by Septin 9 DNA Methylation Assay
Source: PLoS One. 2008 Nov 19;3(11):e3759. doi: 10.1371/journal.pone.0003759 (PMC2582436; doi:10.1371/journal.pone.0003759)
Supplement: Table S1 — Primer, probe and blocker sequences of the marker SEPT9 real-time PCR assay, and the control CFF1 and HB14 real-time PCR assays (0.02 MB DOC) [file pone.0003759.s002.doc]

**Table S1**. Primer, probe and blocker sequences of the marker SEPT9 real-time PCR assay, and the control CFF1 and HB14 real-time PCR assays.

| Assay | Forward Primer | Reverse Primer | Blocker | Probe |
| --- | --- | --- | --- | --- |
| SEPT9 | GtAGtAGttAGtttAGtAtttAttTT | CCCACCAaCCATCATaT | CATCATaTCAaACCCCACAaTCAACACACAaC-C3 | Gttcgaaatgattttatttagttgc-FL  LC-Red640-cgttgatcgcggggttc-PH |
| CFF1 | TAAGAGTAATAATGGATGGATGATG | CCTCCCATCTCCCTTCC | N/A | 6FAM-ATGGATGAAGAAAGAAAGGATGAGT-BHQ-1 |
| HB14 | tggtgatggaggaggtttagtaagt | aaccaataaaacctactcctcccttaa | N/A | FAM-accaccacccaacacacaataacaaacaca-BHQ1a |
