# Supplementary material for: Sensitive Detection of Colorectal Cancer in Peripheral Blood by Septin 9 DNA Methylation Assay
Source: PLoS One. 2008 Nov 19;3(11):e3759. doi: 10.1371/journal.pone.0003759 (PMC2582436; doi:10.1371/journal.pone.0003759)
Supplement: Table S2 — SEPT9 marker performance in training set - alternative algorithms. (0.02 MB DOC) [file pone.0003759.s003.doc]

**Table S2.** SEPT9 marker performance in training set – alternative algorithms.

|  | CRC vs. Normal | |
| --- | --- | --- |
|  | Not Normalized | Normalized |
|  | Sensitivity Specificity | Sensitivity Specificity |
| Qualitative | 48% 93% |  |
| Quantitative | 47% 95% | 47% 95% |
