# Supplementary material for: Sensitive Detection of Colorectal Cancer in Peripheral Blood by Septin 9 DNA Methylation Assay
Source: PLoS One. 2008 Nov 19;3(11):e3759. doi: 10.1371/journal.pone.0003759 (PMC2582436; doi:10.1371/journal.pone.0003759)
Supplement: Table S3 — SEPT9 marker performance in test set - alternative analysis (0.03 MB DOC) [file pone.0003759.s004.doc]

**Table S3.** SEPT9 marker performance in test set – alternative analysis.

33%

23%

Polyps > 1 cm

25%

3%

Polyps < 1 cm

**77%**

**65%**

**Sensitivity (all)**

Sensitivity

(by stage)

**1 out of 3 OR 1 diluted**

**3 out of 3 OR 1 diluted**

Analysis

**75%**

100%

80%

74%

74%

122 / 162

**High sensitivity setting**

**High specificity setting**

120 / 162

Cancers/normals

(total number)

**97%**

100%

66%

69%

40%

IV

**Specificity (set)**

III

II

I
