# Supplementary material for: Sensitive Detection of Colorectal Cancer in Peripheral Blood by Septin 9 DNA Methylation Assay
Source: PLoS One. 2008 Nov 19;3(11):e3759. doi: 10.1371/journal.pone.0003759 (PMC2582436; doi:10.1371/journal.pone.0003759)
Supplement: Figure S1 — Shewhart control charts of total genomic DNA recovery (upper) and SEPT9 marker DNA (lower) for processing controls in the training set. (0.08 MB DOC) [file pone.0003759.s005.doc]

**
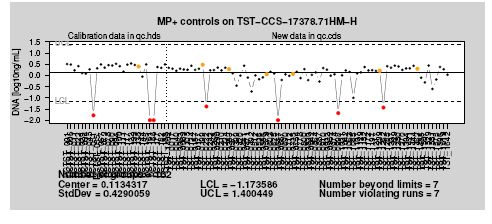
**

**Figure S1.** Shewhart control charts of total genomic DNA recovery (upper) and SEPT9 marker DNA (lower) for processing controls in the training set. The vertical dotted line separates process calibration phase (only controls processed) from measurement phase (controls and clinical samples processed). Note the reduction in total DNA yield in the upper panel.
