# Supplementary material for: Sensitive Detection of Colorectal Cancer in Peripheral Blood by Septin 9 DNA Methylation Assay
Source: PLoS One. 2008 Nov 19;3(11):e3759. doi: 10.1371/journal.pone.0003759 (PMC2582436; doi:10.1371/journal.pone.0003759)
Supplement: Figure S2 — Shewhart control charts of total genomic DNA recovery (upper) and SEPT9 marker DNA (lower) for processing controls in the test set. (0.08 MB DOC) [file pone.0003759.s006.doc]

**Figure S2.** Shewhart control charts of total genomic DNA recovery (upper) and SEPT9 marker DNA (lower) for processing controls in the test set. The vertical dotted line separates process calibration phase (only controls processed) from measurement phase (controls and clinical samples processed). Note negative results in a set of positive controls indicative of inhibition for the SEPT9 marker PCR in the lower panel.
